# Reconsidering the risks of *Linguatula* in Australia: A review of an overlooked introduced parasite

**DOI:** 10.1007/s00436-026-08705-2

**Published:** 2026-06-05

**Authors:** Shokoofeh Shamsi, Diane P. Barton

**Affiliations:** 1 School of Agricultural, Environmental and Veterinary Sciences, Gulbali Institute, Charles Sturt University, Wagga Wagga, Australia; 2 Rural Health Research Institute, Charles Sturt University, Wagga Wagga, Australia

**Keywords:** Emerging infectious diseases, Wildlife–livestock interface, Foodborne parasitic infection, Pentastomida

## Abstract

*Linguatula serrata* is a globally distributed zoonotic parasite that remains largely overlooked in public health. Although traditionally described as having an indirect life cycle involving carnivorous definitive hosts and herbivorous intermediate hosts, emerging evidence suggests greater complexity in transmission pathways and host associations, with some observations raising the possibility of a direct life cycle. In Australia, *L. serrata*, likely introduced through domestic animals and livestock during colonisation, is now well established across both introduced and native hosts, indicating sustained transmission at the wildlife–livestock–human interface, although human infection has not been reported. Recognised transmission pathways, via ingestion of eggs from environmental contamination or infective nymphs in raw or undercooked offal, are plausible in Australia. This Perspective synthesises current knowledge of its biology, transmission, and host range, and highlights its role as both a foodborne and environmentally transmitted zoonosis. We argue that the absence of reported human cases likely reflects under-recognition due to non-specific clinical presentation, diagnostic limitations, and limited awareness among health professionals. Together, these observations suggest that *L. serrata* may be more epidemiologically relevant in Australia than currently recognised and should be reconsidered within Australia’s One Health and zoonotic disease frameworks.

## Highlights

*Linguatula serrata* is likely more widespread in Australia than currently recognised.

The parasite circulates across domestic animals, livestock, feral species, and wildlife hosts.

Reports from native Australian wildlife indicate successful establishment of this introduced parasite in local ecosystems.

Diagnostic challenges and low awareness likely contribute to under-recognition of human infection.

The transmission biology of *Linguatula* spp. may be more flexible than traditionally assumed.

Increased One Health surveillance is needed to clarify the public health significance of *L. serrata*.

## Introduction

Zoonotic parasites continue to present complex challenges at the interface of human, animal, and environmental health, particularly when their presence is underestimated or overlooked (Molyneux et al. 2011; Mubemba et al. 2022). Among these, *Linguatula* spp. represent a striking example of a parasite that is biologically well-established yet epidemiologically neglected. Commonly referred to as “tongue worms”, this name is somewhat misleading: these organisms are neither true worms nor do they infect the tongue. The term instead arises from their elongated, flattened, and annulated body, which superficially resembles a vertebrate tongue. *Linguatula* spp. belong to the Pentastomida, a group occupying a unique and still-debated phylogenetic position (Barton et al. 2022a; Mohanta and Itagaki 2017; Shamsi et al. 2020a, 2022), and have long been recognised for their zoonotic potential (Bhende et al. 2014; Koehsler et al. 2011; Tabibian et al. 2012; Yazdani et al. 2014; Yilmaz et al. 2011).

Specimens of *Linguatula* are remarkedly adept at remaining undetected (Marchetti et al. 2023). This is largely due to their predilection for anatomical sites that are rarely examined during routine necropsies, post-mortem investigations, or abattoir inspections. Nymphal (larval) stages are small, usually less than 1 cm, and are usually found in lymph nodes within the body cavity (Barton et al. 2019; Hobmaier and Hobmaier 1940), whereas the very large adults (often over 10 cm) are found in the upper nasal cavity (Shamsi et al. 2017). This combination of biological distinctiveness and diagnostic invisibility has likely contributed to their continued under-recognition in both veterinary and medical contexts.

Although the genus *Linguatula* comprises multiple species (Christoffersen and De Assis 2013), *Linguatula serrata* is by far the most widely recognised and studied member, and it is this species that forms the primary focus of the present review. Globally, *L. serrata* has been associated with both veterinary and human infections, including nasopharyngeal linguatulosis (Halzoun syndrome) and visceral disease. Despite this, the parasite rarely features in contemporary diagnostic considerations. In Australia, where interactions among wildlife, livestock, companion animals, and humans are frequent and intensifying, such neglect is particularly concerning (Barton et al. 2022b). These interconnected systems, combined with strong reliance on livestock industries and growing awareness of foodborne zoonoses, position Australia as a critical yet underexplored setting for understanding the public and animal health relevance of parasites, particularly *L. serrata*.

This perspective examines the current state of knowledge regarding *Linguatula* in Australia and argues that the parasite’s apparent rarity reflects limited detection rather than absence. By highlighting existing evidence and critical knowledge gaps, we aim to reposition *Linguatula* within Australia’s One Health agenda.

### Life cycle complexity and taxonomic uncertainty in *Linguatula*

The life cycle of *Linguatula serrata* is traditionally considered indirect (Haugerud and Nilssen 1990; Hobmaier and Hobmaier 1940; Sinclair 1954), involving carnivorous definitive hosts and herbivorous intermediate hosts, including livestock (Haugerud and Nilssen 1990; Hobmaier and Hobmaier 1940; Sinclair 1954). Adult parasites inhabit the nasal passages and sinuses of definitive hosts, releasing eggs into the environment via nasal secretions or faeces. Intermediate hosts become infected through ingestion of these eggs, leading to the development of nymphal stages within visceral organs. However, accumulating historical and contemporary evidence suggests that this classical model may not fully capture the diversity of transmission pathways in this genus.

As early as the 20^th^ century, *Linguatula* specimens recovered from the nasopharynx of ruminants such as reindeer and caribou were proposed to represent a distinct species, later recognised as *Linguatula arctica*, largely due to their atypical host association (Chapin 1926; Christensson et al. 1974; Murie 1935; Rehbinder and Nordkvist 1982; Riley 1986; Skjenneberg 1965; Skjenneberg and Slagsvold 1968; Voblikova 1961). Subsequent studies in Scandinavian reindeer populations have consistently reported high prevalences of both adult and pre-adult stages within the nasopharyngeal region of herbivorous hosts, particularly in younger animals (Riley 1986). The repeated detection of adult *Linguatula* in herbivorous mammals challenges the assumption of an obligate predator–prey transmission cycle. In such systems, the involvement of carnivorous definitive hosts is not readily apparent, raising the possibility that transmission does not always conform to the conventional indirect pathway.

This uncertainty is further compounded by unresolved taxonomic issues. Substantial variability between sequences across genetic markers, combined with inaccurate, incomplete or missing morphological verification compound the issues (Barton et al. 2022a; Shamsi et al. 2020b). For example, Barton et al. (2022a) reported pentastomes recovered from roe deer (*Capreolus capreolus*) from Romania, initially presumed to be *L. arctica*, were instead identified as mature adult males of an unspecified *Linguatula* species through morphological assessment. However, molecular sequencing of specimens from the roe deer yielded 18 S rRNA sequences identical to those of *L. serrata*, although Shamsi et al. (2022) showed that 28 S rRNA sequences have greater suitability for differentiation between species of *Linguatula*. This finding shows that the prevailing assumption that adult *Linguatula* infecting herbivores represent *L. arctica* (Gjerde 2013) needs to be treated with caution. Together, these observations point to a critical gap in our understanding of both species boundaries and transmission pathways for species of *Linguatula*.

Historical experimental and observational studies further support the possibility of greater flexibility in the life cycle of *Linguatula* spp. Early work by Leuckart (1860) demonstrated that, under certain conditions, the definitive host may also function as an intermediate host, with larval and nymphal stages developing within visceral lymphatic tissues before migrating to the upper respiratory tract to mature into adults. In such cases, the classical requirement for a separate intermediate host may be bypassed. In addition, nymphal stages may be transferred between hosts without progressing to adulthood, analogous to paratenic transmission described in other parasitic systems. This capacity for host switching may explain the occurrence of *Linguatula* in unexpected or atypical hosts and further complicates interpretation of field observations.

Taken together, the convergence of atypical host associations, unresolved taxonomy, and historical experimental evidence suggests that the life cycle of *Linguatula* spp. may be more plastic than traditionally assumed. Rather than representing a single fixed pathway, transmission may occur along a spectrum of strategies depending on ecological and host-related factors. It is therefore plausible that, at least in some *Linguatula* spp., transmission does not strictly depend on a carnivore–herbivore cycle. Alternative pathways, including the possibility of a direct life cycle or involvement of incidental, herbage-associated invertebrates (Riley 1986), warrant targeted investigation. Although these hypotheses remain to be experimentally validated, they are both biologically plausible and epidemiologically relevant. Resolving these uncertainties is essential for accurate parasite identification, improved surveillance, and a more complete assessment of zoonotic risk within a One Health framework.

### Establishment and transmission of *Linguatula serrata* in Australia

The knowledge on *Linguatula serrata* in Australia has been inferred primarily from historical observations, experimental studies, and sporadic reports of infection in domestic and wild hosts. Available evidence suggests that the parasite was likely introduced into Australia following the introduction of cattle and domestic dogs during the colonial period, most plausibly via infected cattle and/or dogs. This interpretation is consistent with reports from the United Kingdom, where nymphal stages were commonly documented in cattle and less frequently in sheep (Riley et al. 1985; Sinclair 1954).

**Fig. 1:**
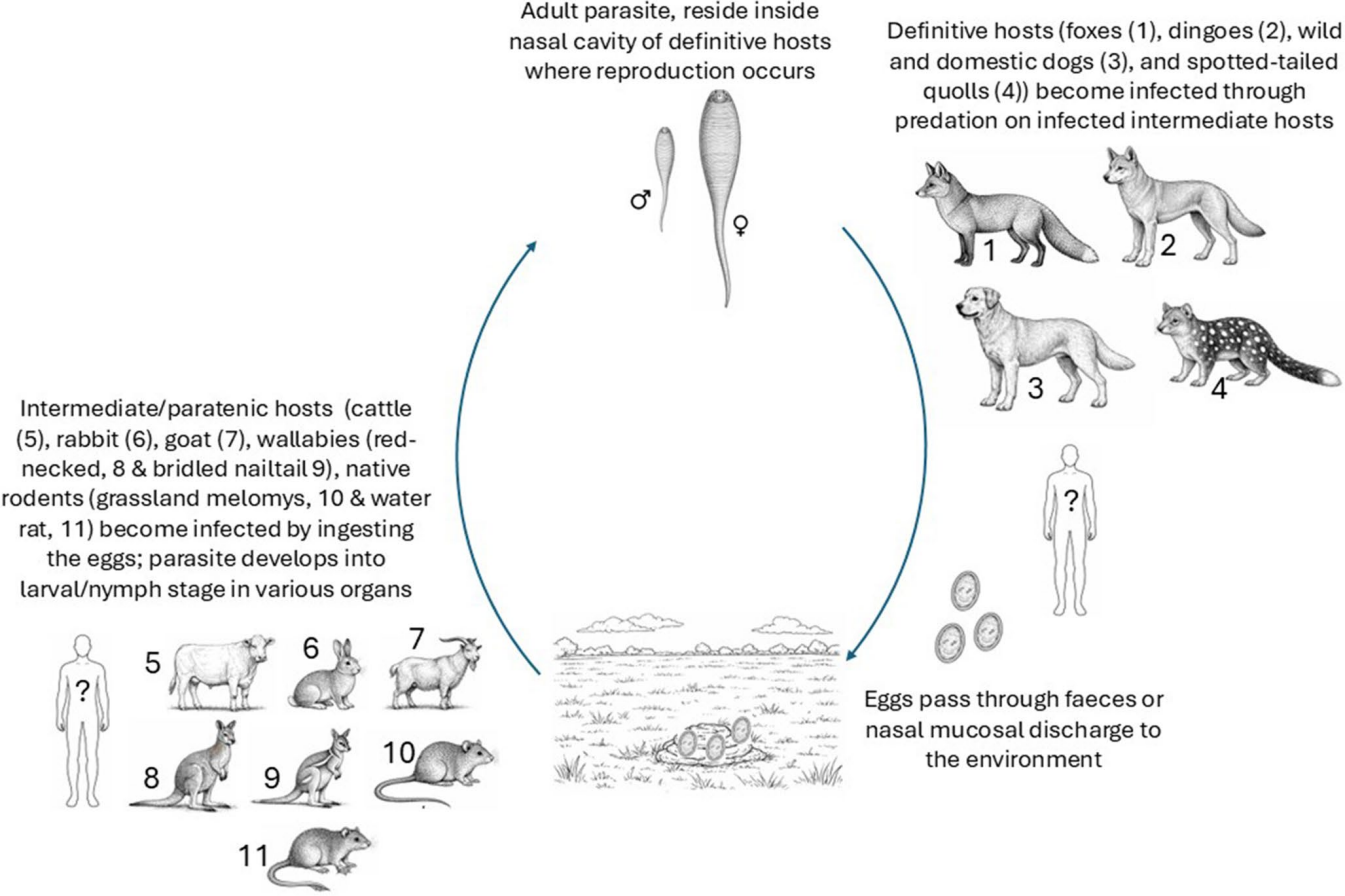
Proposed life cycle of *Linguatula serrata* in Australia. Humans are depicted with a question mark to indicate the absence of confirmed evidence, but possibility, of their involvement in the life cycle in Australia

In Australia, *L. serrata* has been recorded in both introduced and native hosts (Fig. 1), indicating successful establishment within diverse ecosystems. Its capacity to exploit a broad host range across livestock and wildlife highlights considerable ecological adaptability and supports the potential for ongoing transmission at the wildlife–livestock–human interface. The relative scarcity of contemporary reports is therefore unlikely to reflect true absence, but rather limited detection and surveillance.

Many host species recognised elsewhere are also present in Australia, either as domestic animals or feral populations. This overlap suggests that the parasite’s host range may be broader than currently documented. Given that surveillance has been largely opportunistic, additional infected host species are likely to remain undetected. This under-recognition is particularly plausible in wildlife, where routine parasitological examination is uncommon, and in anatomical sites not typically examined during necropsy or meat inspection.

### Life cycle in Australia (Fig. 1)

Summary of reports of *L. serrata* in Australia is provided in Table 1.

**Table 1:** Summary of reports of *L. serrata* in Australia

Host (common name)	Host(scientific name)	Infected organs	Notes	Location	Date occurred	Publication year	Author
Cattle	*Bos taurus*	NS	-	Melbourne, Victoria	1864	1865	Ralph
Cattle	*Bos taurus*	Throughout the body, including the throat, neck, brisket, and intestines.	Many cattle were reported to have swellings and tumours, initially suspected to be tuberculosis and locally referred to as “luinpies.” Tumours and abscesses, ranging in size from a walnut to a coconut. Some lesions were firm, while others had softened into purulent material; in all cases, the contents were enclosed within a well-defined fibrous cyst. These cases were later considered to represent *Linguatula* infection by Johnston and Cleland (1910)	Burnett district, Queensland	1893	1893	Barnard and Park
Cattle (possibly other animals)	*Bos taurus*	NS	Found in 50% of animal slaughtered	Sydney, New South Wales	-	1893	Barnard and Park
Pure country dingo	*Canis lupus dingo*	Nasal cavities	Location not specified in the original report (1910); subsequently reported as South Australia by T. H. Johnston (1916).	South Australia	1904	1910	Johnson
Cattle	*Bos taurus*	Mesenteric glands	-	Illawarra, New South Wales	-	1910	Johnston and Cleland
Dogs	*Canis familiaris*	Nasal cavities	Obtained experimentally (also see Johnston 1916) by introducing the larvae (specimens of which were exhibited) of the parasite, found in the mesenteric glands of cattle from various parts of New South Wales		-	1911	Johnson
Cattle	*Bos taurus*	Mesenteric glands	-	Brisbane abattoirs, Queensland	-	1918	Johnson
Dog	*Canis familiaris*	NS	The dog was obtained from Lost Dogs’ Home	Melbourne University, Victoria	1933	1936	Pullar
Domestic dog	*Canis familiaris*	Expelled from the nose	Reported by a Veterinary Clinic	Melbourne, Victoria	1911	1936	Pullar
Domestic dog	*Canis familiaris*	Expelled from the nose	Adult female was spontaneously expelled from the nostril of a dog; the dog had been introduced into Victoria from Tasmania three years earlier.	Victoria	1935	1936	Pullar
Cattle	*Bos taurus*	NS	Frequently seen in cattle slaughtered in Victoria	Victoria	NS	1936	Pullar
Fox	*Vulpes vulpes*	Nasal cavities	-	Gippsland, Victoria	NS	1946	Pullar
Water rat	*Hydromys chrysogaster*	-	-	North Queensland	-	1958	Mackerras
Grassland Melomys	*Melomys burtoni*	-	-	North Queensland	-	1958	Mackerras
Dingo	*Canis familiaris dingo*	Nasal sinus	-	Dart River, Victoria	NS	1985	Riley et al.
European rabbit	*Oryctolagus cuniculus*	Lung	In the collection of the Queensland Museum (QM; GL11999)	Snowy Plains, New South Wales	NS	1985	Riley et al.
Bridled nailtail wallaby	*Onychogalea fraenata*	-	See Barton et al. 2020	Central Queensland	-	2001	Turni and Smales
Wild dog	*Canis lupus dingo *and* C.l. dingo *x* Canis familiaris*	-	-	Booroomba, Australian Capital Territory	2015	2017	Shamsi et al.
Wild dog	*Canis lupus dingo *and* C.l. dingo *x* Canis familiaris*	-	-	Tumbarumba, New South Wales	2015	2017	Shamsi et al.
Wild dog	*Canis lupus dingo *and* C.l. dingo *x* Canis familiaris*	-	-	Wee Jasper, New South Wales	2015	2017	Shamsi et al.
Wild dog	*Canis lupus dingo *and* C.l. dingo *x* Canis familiaris*	-	-	Mullion, New South Wales	2015	2017	Shamsi et al.
Wild dog	*Canis lupus dingo *and* C.l. dingo *x* Canis familiaris*	-	-	Limestone, New South Wales	2015	2017	Shamsi et al.
Wild dog	*Canis lupus dingo *and* C.l. dingo *x* Canis familiaris*	-	-	Brindabella, New South Wales	2015	2017	Shamsi et al.
Fox	*Vulpes vulpes*	-	-	Bullen Range, Australian Capital Territory	2015	2017	Shamsi et al.
Fox	*Vulpes vulpes*	-	-	Brindabella, New South Wales	2015	2017	Shamsi et al.
Fox	*Vulpes vulpes*	-	-	Mullion, New South Wales	2015	2017	Shamsi et al.
Fox	*Vulpes vulpes*	-	-	Tumbarumba, New South Wales	2015	2017	Shamsi et al.
Cattle	*Bos taurus*	-	-	Corryong, Victoria	2015	2017	Shamsi et al.
Cattle	*Bos taurus*	-	-	Mitta Mitta, Victoria	2015	2017	Shamsi et al.
Cattle	*Bos taurus*	-	-	Tallangatta, Victoria	2015	2017	Shamsi et al.
Cattle	*Bos taurus*	-	-	Tumbarumba, New South Wales	2015	2017	Shamsi et al.
Red-necked wallaby	*Notomacropus rufogriseus*	Liver, Lung	-	Chimney Ridge, New South Wales	2019	2019	Barton et al.
Red-necked wallaby	*Notomacropus rufogriseus*	Liver, Lung	-	Moonbah, New South Wales	2019	2019	Barton et al.
Rabbits	*Oryctolagus cuniculus*	-	-	Chimney Ridge, New South Wales	2020	2020	Barton et al.
Spotted-Tail Quoll	*Dasyurus maculatus*	Nasal cavity	Initially identified as a nymphal stage by Spratt (2002)	Alpine National Park, Victoria	1987	2021	Barton et al.
Feral goats	*Capra hircus*	Mesenteric lymph node	-	Cooma, New South Wales	2020–2021	2022	Barton et al.

#### Definitive hosts

The first report of adult *Linguatula serrata* in Australia described a female specimen recovered from the nasal cavity of a dingo in South Australia, initially classified as a distinct species, *L. dingophila* (Johnson 1910). This classification was subsequently revised, with later studies recognising the specimen as *L. serrata* and attributing the observed morphological differences to developmental stage rather than species-level variation (Johnston 1916).

Experimental studies have provided key insights into transmission pathways. Johnston (Johnston 1911) demonstrated that nymphs recovered from cattle could infect domestic dogs and develop into adult parasites, supporting the classical indirect life cycle involving herbivorous intermediate hosts and carnivorous definitive hosts.

Field observations further support the role of domestic and wild canids in transmission. For example, a spontaneously expelled adult parasite from a domestic dog in Victoria provided direct evidence of infection in definitive hosts (Pullar 1936). Early surveys of fox populations in Victoria reported adult infection rates of approximately 15%, although no infections were detected in domestic dogs in that study (Pullar 1946).

**Fig. 2:**
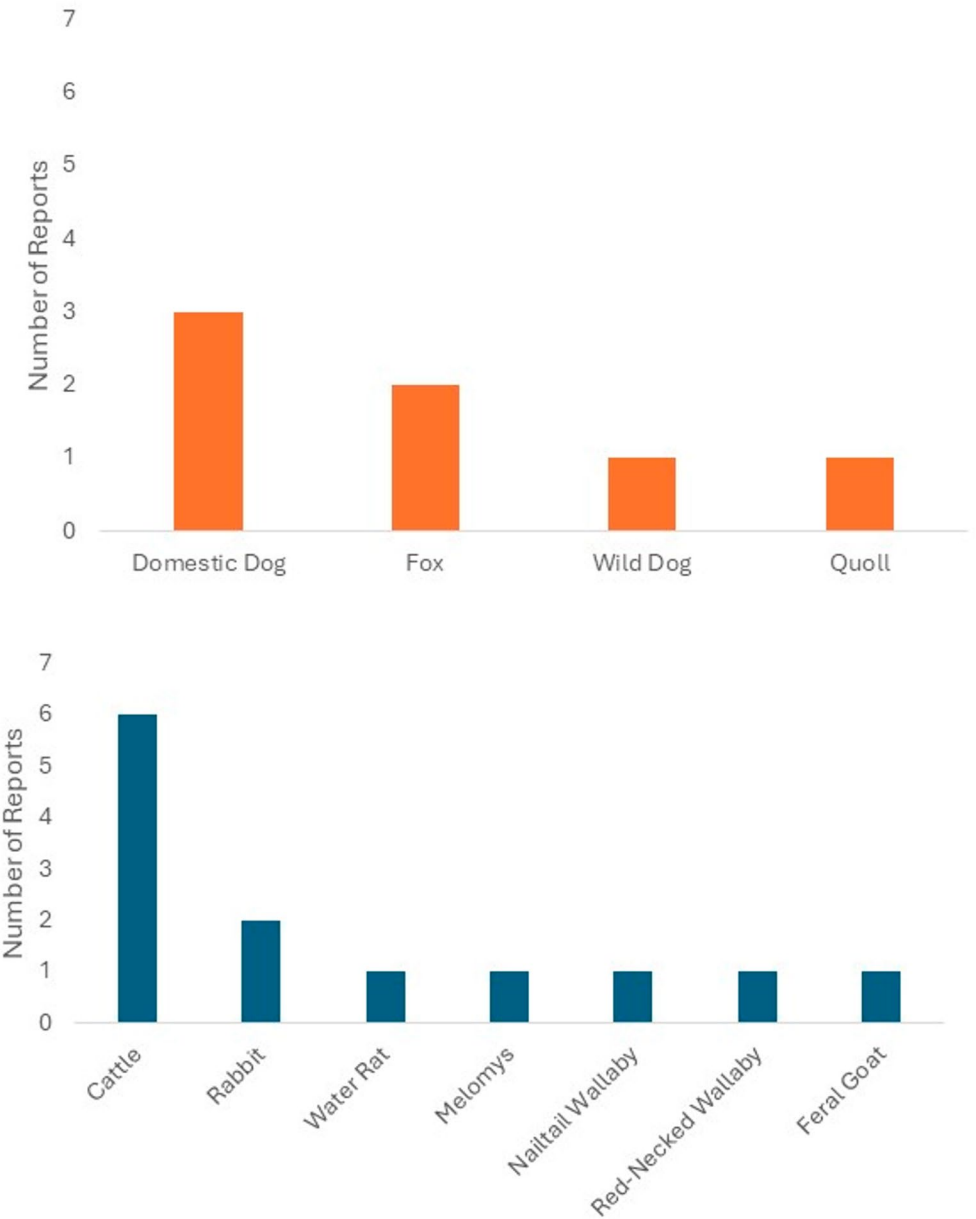
Number of reported records by definitive hosts (top) and intermediate hosts (bottom) in Australia

Subsequent investigations have consistently identified adult *L. serrata* in wild canids, including dingoes in Queensland (Durie and Riek 1952). More recent reports indicate that the parasite continues to circulate across a broad host range in Australia, with adult stages recorded in wild dogs and foxes (Birckhead et al. 2024; Shamsi et al. 2017, 2020a), as well as in a native mammal, the spotted-tailed quoll (Barton et al. 2021) (Fig. 2).

#### Intermediate hosts

The earliest documented record of *Linguatula serrata* in Australia dates to the mid-19^th^ century (Table 2), when nymphs were identified in the mesenteric lymph nodes of cattle in Victoria during post-mortem examinations (Ralph 1865). Subsequent reports from Queensland described encysted parasites within the viscera of cattle, later confirmed as nymphal *L. serrata* (Barnard and Park 1893; Johnston and Cleland 1910). Additional findings from New South Wales further supported the presence of nymphal stages in cattle, particularly within mesenteric lymph nodes. Reports from Queensland abattoirs also confirmed infections in processed cattle (Johnston 1918), reinforcing the role of livestock in maintaining the parasite’s life cycle.

**Table 2:** Timeline of reports of infections with *Linguatula serrata* in animals in Australia

Larval Stage	Year	Adult Stage
Cattle	1864	
Cattle	1893	
	1904	Dingo^1*^
Cattle	1910	
	1911	Domestic Dog
Cattle	1918	
	1933	Domestic Dog
	1935	Domestic Dog
Cattle	1936	
	1946	Fox
Water Rat^1^	1958	
Grassland *Melomys*^1^	1958	
Rabbit	1985	Dingo^1*^
	1987	Spotted-Tail Quoll^1,2^
Bridled Nailtail Wallaby^1^	2001	
Cattle	2015	Wild Dog
	2015	Fox
Red-Necked Wallaby^1^	2019	
Rabbit	2020	
Feral Goat	2020–2021	

^1^Australian native animal. * denotes native to Australia in an ecological and conservation sense, although this host is not indigenous in the strict evolutionary sense because they arrived with humans rather than evolving in Australia.

^2^The parasite in this host was originally identified as a larval stage (Spratt, 2002) but later confirmed to be an adult stage (Barton et al. 2021)

During the first half of the 20^th^ century, nymphs were detected in both cattle and rabbits, leading to the hypothesis of a sylvatic transmission cycle involving foxes and rabbits, with livestock potentially acting as incidental hosts.

Subsequent studies continued to report nymphal stages in cattle in Queensland (Durie and Riek 1952). However, despite indications that *L. serrata* may have been relatively common in certain regions, many of these investigations lacked systematic sampling or quantitative prevalence data, limiting accurate assessment of its distribution and burden.

More recent evidence suggests that the parasite persists across a broad range of intermediate hosts in Australia, including rabbits, red-necked wallabies, Grassland Melomys, goats, and cattle (Barton et al. 2019, 2020, 2021, 2022b; Shamsi et al. 2017), highlighting its ecological flexibility and capacity to exploit both domestic and wildlife systems.

### Human infection and transmission pathways

*Linguatula serrata* is a zoonotic parasite (Barton and Shamsi 2024) capable of infecting humans through multiple exposure pathways. Infection most commonly occurs through ingestion of parasite eggs shed by infected definitive hosts (Abadi et al. 1996; Acha and Szyfres 2003; Attia et al. 2017). Eggs are expelled in nasal secretions and, to a lesser extent, faeces of carnivores such as dogs, foxes, and other canids or felids, contaminating the surrounding environment. Humans may inadvertently ingest these eggs through poor hand hygiene following contact with infected animals, including routine activities such as petting dogs (Abadi et al. 1996; Acha and Szyfres 2003; Attia et al. 2017; Nagamori et al. 2019). Additional exposure pathways include consumption of contaminated food or water, particularly unwashed fruits and vegetables, as well as environmental contact during outdoor activities such as camping, where soil or surfaces may be contaminated. These routes highlight the importance of close human–animal interactions and environmental interfaces in facilitating transmission.

In addition to egg-mediated transmission, human infection may occur through ingestion of infective nymphs present in the tissues of intermediate hosts. This route is typically associated with the consumption of raw or undercooked offal, particularly liver, lymph nodes, or other visceral organs of infected livestock or wildlife (Abadi et al. 1996; Acha and Szyfres 2003; Attia et al. 2017; Nagamori et al. 2019). In such cases, *L. serrata* functions as a foodborne zoonotic parasite, with transmission closely linked to food preparation practices and cultural dietary habits. Following ingestion, nymphs migrate to the nasopharyngeal region, resulting in nasopharyngeal linguatulosis.

Taken together, these transmission pathways indicate that *L. serrata* occupies a dual niche, functioning both as a foodborne parasite and an environmentally transmitted zoonosis. This duality complicates risk assessment and likely contributes to its under-recognition in both clinical and public health settings. The clinical manifestations of infection are determined largely by the developmental stage of the parasite ingested and its subsequent migration within the human host.

#### Visceral linguatulosis

This form arises following ingestion of parasite eggs, typically via contaminated food, water, or hands. Once ingested, larvae hatch in the gastrointestinal tract, penetrate the intestinal wall, and migrate to internal organs where they encyst, most commonly in the liver (Baird et al. 1988), lungs, or lymph nodes. Infections are often asymptomatic or associated with non-specific clinical signs and are frequently detected incidentally during imaging or autopsy (Barton and Shamsi 2024; Tobie et al. 1957). Consequently, the true burden of visceral linguatulosis is likely underestimated, particularly in endemic regions.

#### Nasopharyngeal linguatulosis (Halzoun or Marrara syndrome)

This is the most clinically apparent form of infection (Barton and Shamsi 2024; Tabaripour et al. 2021) and occurs following ingestion of infective nymphs, typically through consumption of raw or undercooked offal. After ingestion, nymphs excyst in the gastrointestinal tract and rapidly migrate via the oesophagus to the nasopharynx, where they attach to the mucosa. This form is considered the most frequently reported manifestation of human linguatulosis globally (Tabaripour et al. 2021), with cases documented from Asia, Africa, as well as sporadic reports elsewhere (Barton and Shamsi 2024; Hajipour and Tavassoli 2019; Tabaripour et al. 2021).

Clinical signs (Morsy et al. 1999; Sadjjadi et al. 1998; Yilmaz et al. 2011) typically develop within hours and include throat irritation, coughing, sneezing, dysphagia, nausea, and respiratory distress. Although symptoms can be acute and severe, they are usually self-limiting. In many cases, paroxysmal coughing or sneezing leads to mechanical expulsion of the parasite and rapid symptom relief. However, severe complications may occur, including airway obstruction or hypersensitivity reactions, particularly in individuals with prior exposure to the parasite (Bowman 1995; Paré 2008; Riley 1986). In rare instances, these infections may be life-threatening.

#### Ocular linguatulosis

In some cases, larvae may migrate to atypical sites, including the eye, resulting in ocular infection (Lazo et al. 1999). This form is uncommon but clinically significant, as it may lead to inflammation, visual disturbance, or damage to ocular structures. Such cases highlight the parasite’s capacity for aberrant migration and the diagnostic challenges it presents. The occurrence of ocular linguatuliasis has been reported in North and South America, Europe and Asia (Anderson and Roberts 1962; Deweese et al. 1962; Hunter and Higgins 1960; Koehsler et al. 2011; Lang et al. 1987; Lazo et al. 1999; Rendtorff and Deweese 1962; Saumya Pal et al. 2011; Sousaefaro and RC 1964; Sulyok et al. 2014; Van Acker et al. 2024).

#### Other atypical presentations

Occasional reports describe larvae in unusual anatomical locations, including heart, the bloodstream or other tissues (Abadi et al. 1996; Riley 1986; Zoonoses and Communicable Diseases Common to Man and Animals (3rd ed.) 2003). While uncommon, these presentations further illustrate the biological flexibility of the parasite and the potential for diverse clinical outcomes.

Across all forms, humans are considered accidental hosts. While *L. serrata* can establish and migrate within the human body, there is limited evidence that it completes its full development to reproductively mature adults. Nevertheless, the ability of infective stages to reach sites typically associated with definitive hosts underscores important gaps in our understanding of host specificity and transmission biology.

### Human infection with *L. serrata* in Australia

Despite the well-established zoonotic potential of *L. serrata* and its documented presence across a wide range of animal hosts in Australia (see above), there are currently no published reports of human infection from Australia. However, several lines of evidence suggest that human infection may occur occasionally but remain unrecognised.

First, *L. serrata* is more widely distributed among both domestic and wild animal hosts in Australia, including dogs, foxes, and a variety of intermediate hosts, than previously considered (Shamsi et al. 2017). This potential transmission network creates ongoing opportunities for human exposure, particularly at the wildlife–livestock–human interface. A high infection rate (for example, 67% of wild dogs and 25% of foxes (Shamsi et al. 2017)) has been reported in parts of south-eastern Australia, including regions frequently used for recreational activities such as camping. This region includes significant levels of livestock production, accompanied by working dogs. Farm feeding practices may also contribute to the maintenance of *L. serrata* transmission cycles. In particular, feeding dogs and cats raw or undercooked offal from livestock or wildlife can facilitate infection of definitive hosts, enabling completion of the parasite’s life cycle. Infected animals may subsequently shed eggs into the environment via nasal secretions, increasing the risk of human exposure within domestic settings. As raw feeding practices and home processing of animal products become more common, this pathway may represent an under-recognised contributor to parasite transmission.

Second, known transmission pathways are not unique to endemic regions. Humans may be exposed to infective eggs through routine contact with domestic animals, environmental contamination, or poor hand hygiene, as well as through consumption of contaminated food or water. In addition, ingestion of infective nymphs via raw or undercooked offal represents a recognised foodborne route. Many of the intermediate hosts identified in Australia, including livestock and wildlife species, are also consumed as food. Importantly, infective stages are often located in visceral tissues such as liver, lungs, and lymph nodes, which may be consumed directly or handled during food preparation, increasing the likelihood of exposure.

Australia’s multicultural society and diverse food landscape further broaden this risk. Dishes involving raw or lightly cooked offal, traditionally associated with specific regions, are widely available and increasingly consumed across different cultural groups. As such, potential exposure is not confined to particular communities but may occur more broadly through shared culinary practices. Additionally, the increased consumption of wild-caught animals across sectors of Australian society may increase exposure to parasitic infection (Barton et al. 2023).

Third, clinical recognition of linguatulosis is inherently challenging. Symptoms are often non-specific and may be transient, particularly in nasopharyngeal infections where parasites can be expelled spontaneously. Visceral infections are frequently asymptomatic and may go undetected entirely, meaning cases may not present to healthcare systems or may be misdiagnosed.

Fourth, diagnostic limitations further contribute to under-recognition. There are no widely available serological tests, and identification relies on direct observation or specialist expertise. The unusual morphology and infection location of the parasite may also complicate recognition or lead to misidentification.

A further factor likely contributing to the under-recognition of *L. serrata* infection in Australia is the declining emphasis on parasitology within medical education (Seal et al. 2020). In many medical curricula, parasitology is now integrated into broader infectious disease teaching or reduced in scope, with limited dedicated coverage of less common or regionally overlooked parasites. While this reflects changing priorities in medical training, it may inadvertently reduce clinicians’ familiarity with atypical or emerging parasitic infections, including those with zoonotic potential.

This reduced exposure has practical implications for clinical recognition and diagnosis. Conditions such as linguatulosis, which may present with non-specific or transient symptoms, are unlikely to be considered in differential diagnoses if awareness is low. In addition, the unusual morphology and life cycle of *Linguatula* spp. further complicate identification in the absence of prior training. In a country such as Australia, where the parasite is present in animal hosts but rarely reported in humans, this gap in awareness may contribute to a cycle of under-detection, under-reporting, and continued neglect. Reinforcing parasitology awareness within medical and allied health training may therefore be critical for improving detection of neglected zoonotic infections in Australia.

Taken together, these factors suggest that the apparent absence of human infection in Australia is more likely due to under-detection than true absence. Increasing awareness among clinicians, veterinarians, and the public, alongside improved surveillance and diagnostic capacity, will be essential to determine the true burden and public health significance of this neglected zoonosis.

## Conclusion

*Linguatula serrata* is an overlooked parasite that remains largely absent from contemporary clinical and public health discourse despite its established presence in Australia. Its misleading common name, “tongue worm”, together with its occurrence in anatomical sites not routinely examined during necropsies or meat inspection, likely contributes to its under-recognition. Available evidence suggests that the transmission biology of *Linguatula* spp. is likely more complex and flexible than the classical indirect life cycle traditionally described. Combined with unresolved taxonomic boundaries, these uncertainties complicate interpretation of host associations, transmission dynamics, and zoonotic risk. At the same time, historical and contemporary evidence indicates that *L. serrata* is well established within Australia, circulating among domestic animals, livestock, feral species, and wildlife hosts. Its broad host range and ecological adaptability support the existence of ongoing transmission at the wildlife–livestock–human interface, while the relatively limited number of reports likely reflects under-detection rather than rarity. Globally, *L. serrata* is recognised as a zoonotic parasite capable of infecting humans through both environmental and foodborne pathways, producing a spectrum of clinical manifestations ranging from asymptomatic visceral infection to acute nasopharyngeal disease. The diversity of transmission routes, combined with non-specific or transient clinical presentations and limited diagnostic familiarity, likely contributes to substantial under-recognition of human linguatulosis worldwide. Although no confirmed human infections have been reported from Australia, biologically plausible pathways for human exposure clearly exist. However, the true extent of human infection in Australia remains unknown due to limited surveillance, diagnostic challenges, and the absence of targeted epidemiological investigations. Addressing these knowledge gaps will require increased awareness among clinicians, veterinarians, diagnosticians, and public health professionals, together with integrated morphological, molecular, ecological, and epidemiological investigations within a One Health framework.

## Data Availability

No datasets were generated or analysed during the current study.
